# Gait‐Related Metabolic Covariance Networks at Rest in Parkinson's Disease

**DOI:** 10.1002/mds.28977

**Published:** 2022-03-14

**Authors:** Hilmar P. Sigurdsson, Alison J. Yarnall, Brook Galna, Sue Lord, Lisa Alcock, Rachael A. Lawson, Sean J. Colloby, Michael J. Firbank, John‐Paul Taylor, Nicola Pavese, David J. Brooks, John T. O'Brien, David J. Burn, Lynn Rochester

**Affiliations:** ^1^ Translational and Clinical Research Institute, Faculty of Medical Sciences Newcastle University Newcastle upon Tyne United Kingdom; ^2^ Newcastle upon Tyne Hospitals NHS Foundation Trust Newcastle upon Tyne United Kingdom; ^3^ Health Futures Institute Murdoch University Perth Australia; ^4^ Auckland University of Technology Auckland New Zealand; ^5^ Department of Nuclear Medicine and PET Aarhus University Hospital Aarhus Denmark; ^6^ Department of Psychiatry University of Cambridge Cambridge United Kingdom; ^7^ Faculty of Medical Sciences Newcastle University Newcastle upon Tyne United Kingdom

**Keywords:** [^18^F]‐2‐fluoro‐2‐deoxyglucose, gait, multivariate covariance networks, Parkinson's disease, PET

## Abstract

**Background:**

Gait impairments are characteristic motor manifestations and significant predictors of poor quality of life in Parkinson's disease (PD). Neuroimaging biomarkers for gait impairments in PD could facilitate effective interventions to improve these symptoms and are highly warranted.

**Objective:**

The aim of this study was to identify neural networks of discrete gait impairments in PD.

**Methods:**

Fifty‐five participants with early‐stage PD and 20 age‐matched healthy volunteers underwent quantitative gait assessment deriving 12 discrete spatiotemporal gait characteristics and [^18^F]‐2‐fluoro‐2‐deoxyglucose‐positron emission tomography measuring resting cerebral glucose metabolism. A multivariate spatial covariance approach was used to identify metabolic brain networks that were related to discrete gait characteristics in PD.

**Results:**

In PD, we identified two metabolic gait‐related covariance networks. The first correlated with mean step velocity and mean step length (*pace gait network*), which involved relatively increased and decreased metabolism in frontal cortices, including the dorsolateral prefrontal and orbital frontal, insula, supplementary motor area, ventrolateral thalamus, cerebellum, and cuneus. The second correlated with swing time variability and step time variability (*temporal variability gait network*), which included relatively increased and decreased metabolism in sensorimotor, superior parietal cortex, basal ganglia, insula, hippocampus, red nucleus, and mediodorsal thalamus. Expression of both networks was significantly elevated in participants with PD relative to healthy volunteers and were not related to levodopa dosage or motor severity.

**Conclusions:**

We have identified two novel gait‐related brain networks of altered glucose metabolism at rest. These gait networks could serve as a potential neuroimaging biomarker of gait impairments in PD and facilitate development of therapeutic strategies for these disabling symptoms. © 2022 The Authors. *Movement Disorders* published by Wiley Periodicals LLC on behalf of International Parkinson and Movement Disorder Society

## 1 Introduction

Gait difficulties are common in Parkinson's disease (PD)^1^, ^2^, ^3^ and cause significant disability. They are associated with increased risk of falls^4^ and reduced quality of life. No specific treatments are currently available for these symptoms that become progressively worse with disease severity despite optimal medication.^1^, ^3^, ^5^ Dopamine replacement therapies (DART) form the mainstay of treatment and provide transitory improvements to selected gait characteristics, such as gait velocity,^6^ but can worsen others, such as gait variability.^7^ Due to the limited response to dopaminergic drugs, other systems influencing the neural control of walking in PD have been proposed.^5^, ^8^, ^9^, ^10^, ^11^ In addition, cognitive impairments have been shown to be associated with early gait deficits in patients.^12^

Recent studies have demonstrated that gait impairment is also apparent in the prodromal stages of PD,^13^ leading to an interest in the potential of gait characteristics as biomarkers for early disease identification and monitoring.^5^, ^13^ Despite this, neural mechanisms underlying different gait parameters remain elusive, and dependable neuroimaging biomarkers for discrete gait problems in this disorder are needed. Although gait velocity can be used as a global measure of gait integrity, conceptual gait models have been crafted,^14^, ^15^ and recent work has demonstrated that discrete gait characteristics contained within different domains of gait (eg, pace and variability) are related to selective brain regions and networks in healthy older adults.^16^, ^17^, ^18^ In PD, it is unclear, however, whether there are discrete neural networks that map to specific gait characteristics such as gait variability, asymmetry, or those related to postural control, and how these are affected in people with PD.

[^18^F]‐2‐fluoro‐2‐deoxyglucose‐positron emission tomography (FDG‐PET) and a scaled subprofile model/principal components analysis (SSM/PCA), a multivariate spatial covariance pattern analysis, have been used to successfully derive PD metabolic profiles associated with motor impairment,^19^ tremor,^20^ and cognition^21^ that appear to be modulated by therapeutic intervention. It has therefore been suggested that the quantification of treatment‐mediated changes in these metabolic profiles could potentially provide an objective outcome measure when testing the effects of novel antiparkinsonian therapies.^20^

In this prospective study, we use FDG‐PET and the SSM/PCA approach to identify independent gait‐related brain networks that are altered in people with PD by localizing metabolic changes across brain areas that correlated with specific parameters of gait.^22^ We hypothesized that: (1) gait control is subserved by discrete metabolic gait networks that are independently related to different gait outcomes, and (2) expression of these metabolic gait networks is different in people with PD with gait problems.

## 2 Materials and Methods

### 2.1 Participants

A total of 158 recently diagnosed idiopathic PD participants and 99 healthy volunteers (HVs) were recruited onto the Incidence of Cognitive Impairment in Cohorts with Longitudinal Evaluation (ICICLE)‐PD study. Participants were optionally invited to take part in the collaborative ICICLE‐GAIT study. The details of these two studies have been presented extensively elsewhere.^1^, ^23^ In brief, patients were recruited from community and outpatient clinics in the Newcastle upon Tyne and Gateshead areas of the United Kingdom. A diagnosis of PD was made at baseline by a movement disorder specialist using the Queen's Square Brain Bank Criteria^24^ and confirmed at follow‐up visits every 18 months. For the ICICLE‐GAIT study, participants unable to walk unassisted for a minimum period of 2 minutes or who presented with cognitive impairment (Mini‐Mental State Examination [MMSE] score < 24 or a diagnosis of dementia), mood, or unrelated movement disorder were excluded. This study includes a subset of participants with PD (n = 55/158) who completed both FDG‐PET and gait assessments at study intake. In addition, gait data and FDG‐PET were available from a matched comparator group of 20 HVs. The clinical characteristics of these participants have been previously reported, where the relationship between regional metabolic glucose uptake and cognition was investigated.^25^

The study was approved by the Newcastle and North Tyneside Research Ethics Committee (REC no. 09/H0906/82 and 08/H0906/147) and conducted according to the Declaration of Helsinki. All participants provided written informed consent.

All participants with PD were scanned while in an ON motor state (~1 hour after DART). Global cognition was assessed using the Montreal Cognitive Assessment^26^; severity of locomotor deficits in participants with PD was rated using the Hoehn and Yahr scale^27^ and the Movement Disorder Society Unified Parkinson's Disease Rating Scale part III (MDS‐UPDRS III). Levodopa equivalent daily dose (LEDD) was calculated as previously described.^28^ Motor phenotype status was based on the definition by Stebbins et al^29^ and is summarized in Table 1 for descriptive purposes. This shows that our sample of participants with PD consists of mixed phenotypes. Multimorbidity was defined using the age‐adjusted Charlson Comorbidity Index and the International Classification of Primary Care‐2 conditions as previously reported.^30^ Finally, the anticholinergic burden was computed using the Anticholinergic Drug Scale (ADS),^31^ as previously described.^32^

**TABLE 1:** Demographic and clinical data

Variable (unit or maximum score)	HVs (n = 20)	Participants with PD (n = 55)	*t*/X^2^	*P*
Age (y)	71.62 ± 9.60	73.81 ± 4.95	1.30	0.20
Sex (M/F)	60% male (12 M/8F)	71% male (39 M/16F)	0.80	0.37
Mass (kg)	77.22 ± 11.43	78.20 ± 13.69	0.28	0.78
Height (m)	1.69 ± 0.10	1.69 ± 0.06	0.24	0.81
BMI (kg/m^2^)	26.47 ± 4.01	27.17 ± 3 0.83	0.69	0.49
MoCA (30)^a^	27.4 ± 2.62	24.17 ± 3.73	3.56	<0.001
Age‐adjusted CCI (24)	0.55 ± 1.76	1.33 ± 2.00	0.08	0.94
No. of comorbidities^b^	2 ± 1.49	2.65 ± 1.92	0.13	0.89
No. of medications^c^	2.3 ± 2.64	5.47 ± 2.85	2.51	0.01
Gait to PET scan (mo)	1.95 ± 2.40	−0.77 ± 2.27	–	–
Disease duration^d^ (mo)	–	6.35 ± 4.93	–	–
LEDD (mg/d)	–	170.38 ± 131.93	–	–
Anticholinergic burden (3)	0.15 ± 0.37	0.56 ± 1.01	1.99	0.08
MDS‐UPDRS III score (132)	–	23.87 ± 8.57	‐	**‐**
Hoehn & Yahr stage (V), n (%)				
Stage I	–	11 (20)	–	–
Stage II	–	33 (60)	–	–
Stage III	–	11 (20)	–	–
Motor phenotype (3), n (%)				
Postural instability gait disorder		26 (47.3)		
Tremor dominant		19 (34.5)		
Indeterminate		10 (18.2)		

Values are mean ± 1 SD for continuous variables and frequency distribution for categorical variables. Numbers in parentheses next to variable names indicate maximum possible score for that measure or the unit of measurement.

^a^ MoCA scores were missing for one participant with PD.

^b^ Disease count based on the International Classification of Primary Care‐2 conditions.

^c^ Sum of total prescribed and non‐PD medications.

^d^ Time from diagnosis.

HV, healthy volunteer; PD, Parkinson's disease; M, male; F, female; BMI, body mass index; MoCA, Montreal Cognitive Assessment; CCI, Charlson Comorbidity Index; PET, positron emission tomography; LEDD, levodopa equivalent daily dose.

### 2.2 Gait Assessment

**FIG 1:**
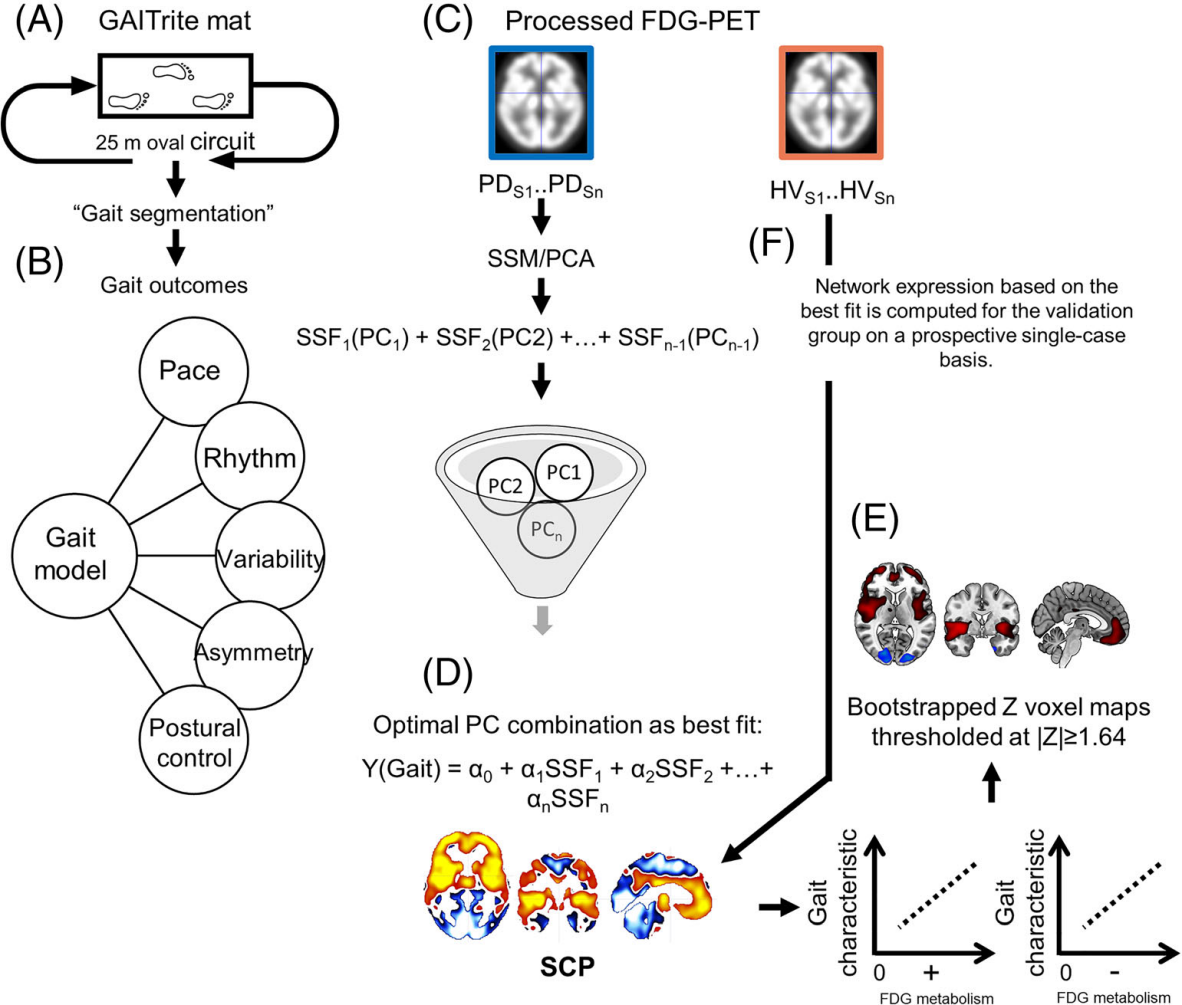
Analysis workflow. Simplified schematic of the data analysis workflow to generate gait‐related metabolic covariance networks in PD. DLPFC, dorsolateral prefrontal cortex; FDG, [^18^F]‐2‐fluoro‐2‐deoxyglucose; HV, healthy volunteers; MCC, mid‐cingulate cortex; OFC, orbital frontal cortex; PC, principal component; PD, Parkinson's disease; PET, positron emission tomography; SCP, spatial covariance pattern; SSF, subject scaling factor; SSM/PCA, scaled subprofile model/principal components analysis; VL, ventrolateral. [Color figure can be viewed at wileyonlinelibrary.com]

All participants completed a comprehensive gait assessment described extensively elsewhere.^1^, ^3^ In brief, participants walked at their preferred speed for 2 minutes around a 25‐m oval circuit (Fig. 1A). Gait was measured using an instrumented mat with embedded pressure sensors (Platinum model GAITRite, dimensions: 7.0 × 0.6 m, sampling frequency: 240 Hz). Based on past findings showing gait impairments in PD (from the same ICICLE‐GAIT cohort),^1^ we derived 12 (of the original 16) spatiotemporal gait characteristics, which have shown to be significantly altered in PD, as components of a validated model that is composed of five gait domains (pace, rhythm, variability, asymmetry, and postural control)^33^ using bespoke algorithms (Fig. 1B).^1^, ^15^

### 2.3 Imaging Acquisition

FDG‐PET images were acquired using a Siemens Biograph 40 Truepoint PET‐CT 30 minutes after an intravenous bolus administration of 250 MBq ^18^F‐FDG. The Siemens scanner software was used for iterative reconstruction (OSEM2D, 6 iterations, 16 subsets) with a tissue attenuation correction based on the CT scan obtained immediately before the FDG‐PET scan. All participants were asked to arrive in a fasting state (at least 4 hours before FDG administration). Blood glucose was measured and was <180 mg/dL (10 mM/L) in all participants.

### 2.4 Processing of FDG‐PET Images

Reconstructed static FDG‐PET acquisitions were preprocessed using SPM12 (7219; Wellcome Trust Centre for Neuroimaging, http://www.fil.ion.ucl.ac.uk) running on Matlab (r2018a; The MathWorks Inc., Natick, MA, USA). All FDG‐PET images were spatially normalized to an age‐appropriate template in Montreal Neurological Institute (MNI) space^34^ using the ‘Old Normalisation’ module in SPM12. The spatially normalized FDG images were then smoothed with an 8‐mm full‐width at half maximum Gaussian kernel. Image quality was good for all participants, resulting in no exclusions.

### 2.5 Network Analysis

To derive gait‐specific spatial covariance networks and characterize these network topographies in people with PD (Fig. 1), we used the SSM/PCA approach by using procedures from the gcva‐pca toolbox (https://www.nitrc.org/projects/gcva_pca).^35^ The spatial covariance gait networks were constructed based on PD FDG‐PET images alone. Although entering images from both groups simultaneously into the algorithm may be advantageous for diagnostic discrimination purposes, it would also lead to more complex and circular analyses. In addition, because the underlying neural systems of gait in PD and typical aging may be different, we believed that the groups should be separated to determine these networks. First, the FDG‐PET scans of participants with PD were restricted to gray matter using an age‐appropriate binary mask (including brainstem, excluding voxels in white matter and cerebrospinal fluid) generated from thresholded and averaged group images. Second, the variance of global mean was removed from the images before being entered into a principal components (PC) analysis generating *N −* 1 PC images (Fig. 1C). This ensured that the resulting components of covarying metabolic glucose consumption with gait parameters were not confounded by individual differences in global FDG uptake.^22^ Third, a one‐dimensional subject‐scaling factor (SSF_FDG_) for expression of each PC was computed where a greater participant‐specific SSF indicates stronger expression of that PC. Fourth, gait‐specific profiles were determined using a multiple linear regression where discrete gait characteristics were entered consecutively as the dependent variable and PC‐specific SSF_FDG_ scores as predictors. The analysis was restricted to PC images 1–10, which accounted for ~75% of the total subject × voxel variance. Model selection of the best fit for each gait profile, which could include single, contiguous, or noncontiguous components, representing a pattern for that specific gait characteristic, was based on the relative goodness of fit as determined by the Akaike Information Criterion (Fig. 1D).

Each PC generated is composed of voxels with either positive or negative weightings, indicating the direction and strength of covariance between voxels. Our data were interpreted in accordance with recent work using a similar approach.^18^, ^36^ As such, positively (greater FDG metabolism) and negatively (reduced FDG metabolism) weighted regions were interpreted as those that have relatively raised and reduced FDG metabolism associated with, for example, greater gait velocity (ie, faster gait). Importantly, in terms of gait variability where higher values indicate greater gait variability, both positively and negatively weighted regions are associated with worsening gait variability.

Finally, we used a bootstrap test (with 1000 repetitions) to measure the reliability of voxel weights for each gait parameter profile as the ratio of voxel weights and bootstrap standard deviation. The outcome is a *z* score voxel map of FDG hypometabolism and hypermetabolism containing brain areas contributing to the covariance pattern with high confidence. The *z* voxel maps were thresholded at |*z*| ≥ 1.64 (Fig. 1E), to exclude voxels with minimal contribution to the overall network, equating to an approximate *P* < 0.05 (one‐tailed) and labeled using the AAL3.1 atlas.^37^ The *P* value for the behavioral fit (*R* ^2^) was obtained with 1000 permutations.

### 2.6 Covariance Pattern Validation

To validate the expression of the PD gait‐related profiles (PDGP) as a marker of altered gait parameters in PD, we quantified the degree of expression of PDGPs (as an SSF_FDG_) in 20 HVs. The one‐dimensional SSF_FDG_s were computed for this group using a voxel‐based automated algorithm on a prospective single‐case basis (Fig. 1F)^38^ from the best fit of PCs for each gait parameter network. SSF_FDG_ scores from patients were standardized to HVs SSF_FDG_.

### 2.7 Statistical Analysis

All statistical analyses were performed using Matlab with a *P* < 0.05 threshold for statistical significance. A correction for multiple comparisons was applied using the false discovery rate. Raw *P* values are reported in the text and tables with an indication if *P* values are less than false discovery rate critical *P*. Differences in gait characteristics were assessed using multiple linear regression with a discrete gait characteristic as the dependent variable and groups as a predictor of interest. Differences in regional covariance network SSF_FDG_ expressions between the two groups were examined using multiple linear regression with SSF_FDG_ as the dependent variable (outcome) and group as a predictor of interest. Regression diagnostics were examined to ensure that the assumptions of linear regression were met, with particular attention to collinearity (variance inflation factor) and influential cases using Cook's distance (Cook's *d*). To evaluate the relationship between network expression scores and PD clinical data (disease duration [time from diagnosis], LEDD, total MDS‐UPDRS III scores, and anticholinergic burden) in PD participants, we used Pearson's partial correlation. For all analyses, age, sex, and Montreal Cognitive Assessment were entered as covariates of no interest. Differences in demographic and anthropometric data were assessed using independent sample *t* tests and chi‐squared (χ^2^) tests as appropriate. Gait variability and asymmetry were nonnormally distributed and transformed using logarithmic and square root transformations, respectively.

## 3 Results

### 3.1 Group Characteristics

Group characteristics are summarized in Table 1. Five patients were not taking any DART medication. Four other patients were taking anticholinergic medication (amitriptyline 10–20 mg every day [n = 3], orphenadrine 50 mg three times per day [n *=* 1]) in addition to receiving DART. The anticholinergic burden, computed using the ADS, indicated that all participants receiving anticholinergic medication were prescribed low doses.

### 3.2 Group Differences in Gait Characteristics

Relative to HVs, participants with PD demonstrated slower gait speed, took shorter steps, and had increased swing time variability, step time variability, and stance time variability (Table 2). No other gait characteristic was significantly different between the groups.

**TABLE 2:** Comparison of discrete gait characteristics between groups

Gait characteristics	HVs (n = 20)	Participants with PD (n = 55)	*t*	*P* ^a^
Mean step velocity (m/s)	1.33 ± 0.260	1.11 ± 0.221	−3.11	**0.003**
Mean step length (m)	0.71 ± 0.103	0.61 ± 0.092	−3.21	**0.002**
Swing time variability (ms)	2.54 ± 0.290	2.84 ± 0.347	2.51	**0.014**
Mean step time (ms)	538 ± 46	560 ± 50	1.60	0.113
Mean stance time (ms)	685 ± 77	731 ± 81	1.85	0.068
Step length variability (m)	0.02 ± 0.005	0.02 ± 0.008	0.94	0.348
Step time variability (ms)	2.59 ± 0.306	2.87 ± 0.349	2.53	**0.014**
Stance time variability (ms)	2.77 ± 0.354	3.05 ± 0.418	2.44	**0.017**
Step time asymmetry (ms)	3.16 ± 1.404	3.56 ± 2.000	0.66	0.509
Swing time asymmetry (ms)	2.89 ± 1.348	3.08 ± 1.600	0.52	0.607
Stance time asymmetry (ms)	2.78 ± 1.540	3.05 ± 1.494	0.83	0.411
Step width variability (m)	0.02 ± 0.006	0.02 ± 0.007	−1.21	0.232

Values are means ± 1 SD.

^a^ Statistically significant differences between groups after false discovery rate (FDR) correction are shown in boldface (*P* value less than FDR critical *P*).

### 3.3 Quantifying Discrete Metabolic Gait Covariance Networks

The five gait characteristics showing statistically significant differences in PD compared with HVs (mean step velocity, mean step length, swing time variability, step time variability, and stance time variability) were entered into the SSM/PCA analysis to generate PDGPs.

**FIG 2:**
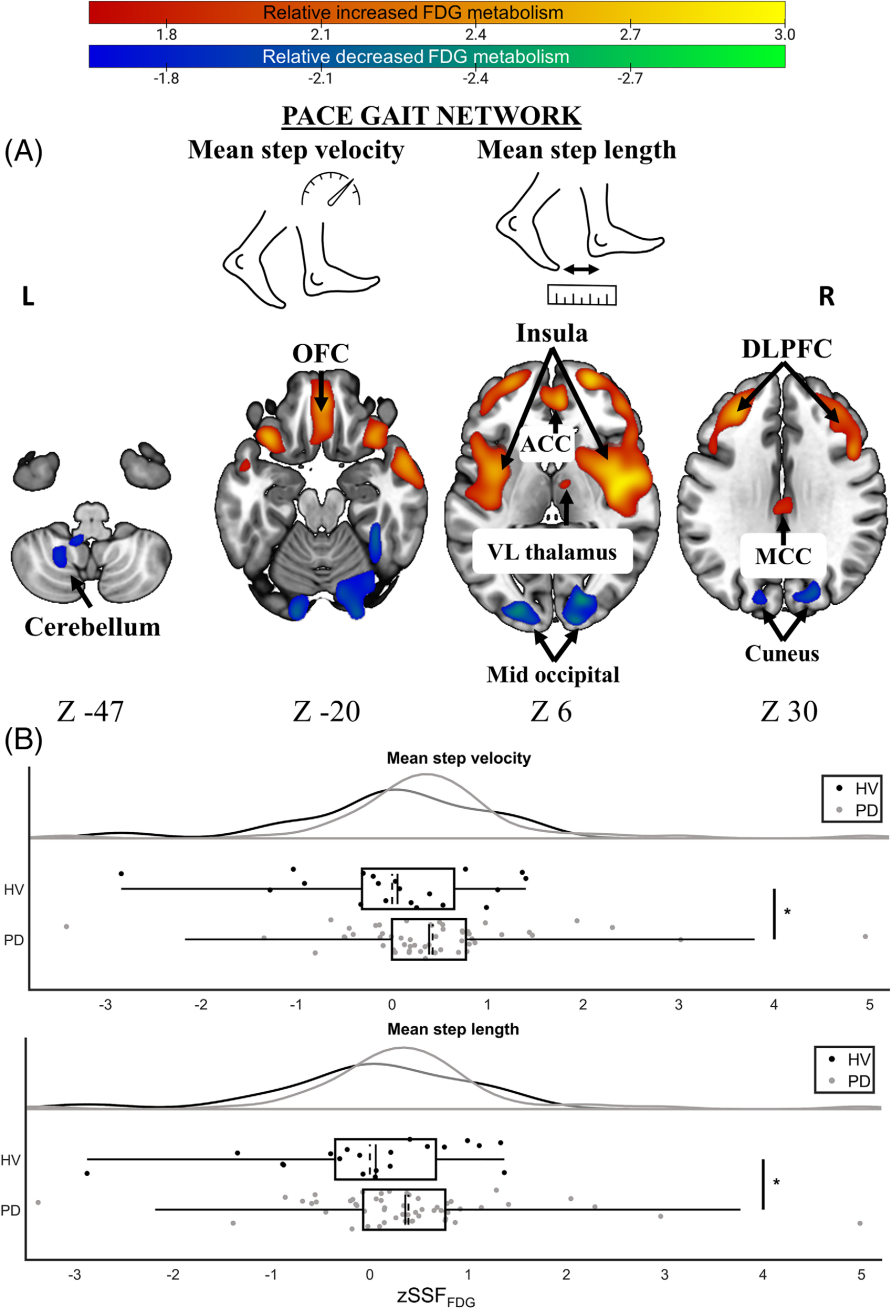
The pace gait network. (**A**) The thresholded *z* voxel map is projected onto the ICBM152 template. Increased and decreased FDG metabolism is shown in hot and cold colors, respectively. (**B**) Distribution of *z* scored SSF_FDG_ for each group and discrete gait characteristics contained within this gait network. Greater zSSF_FDG_ (*x*‐axis) refers to greater network expression. *P < 0.05 corrected for multiple comparisons. Box and whiskers represent the interquartile range. Solid line represents the median, while the broken line is the mean. ACC, anterior cingulate cortex; DLPFC, dorsolateral prefrontal cortex; FDG, [^18^F]‐2‐fluoro‐2‐deoxyglucose; HV, healthy volunteers; MCC, mid‐cingulate cortex; OFC, orbital frontal cortex; PD, Parkinson's disease; SSM/PCA, scaled subprofile model/principal components analysis; VL, ventrolateral; zSSF_FDG_, standardized subject scaling factor. [Color figure can be viewed at wileyonlinelibrary.com]

*Mean step velocity* (*R* ^*2*^ = 0.11, *P* = 0.044) and *mean step length* (*R* ^*2*^ = 0.17, *P* = 0.007) were predicted by a brain network that we refer to as a *pace gait network* (Fig. 2A, linear combination of PCs 2 and 3). The topography of the pace gait network was characterized primarily by relatively increased FDG metabolism in covarying clusters encompassing bilateral superior, middle (including dorsolateral prefrontal cortex [DLPFC]), and inferior frontal gyri; supplementary motor area (SMA); orbital frontal cortex (OFC, including gyrus rectus); anterior, middle, and posterior cingulate cortices; bilateral insula; postcentral gyrus that included the right anterior‐subcentral gyrus; and right ventrolateral thalamus. This relatively increased FDG metabolism was associated with concurrent decreased FDG metabolism in the bilateral lower limb region of the paracentral lobule, left middle temporal gyrus, bilateral fusiform gyri, left middle occipital cortex, and bilateral cerebellum. Anatomical details of the *pace gait network* are summarized in Supporting Information Table S1. The linear combination of PCs 2 and 3 also predicted *stance time variability*; however, the *R* ^2^ value was poor (*R* ^*2*^ = 0.09) and statistically nonsignificant (*P* > 0.05) and was thus not considered further.

**FIG 3:**
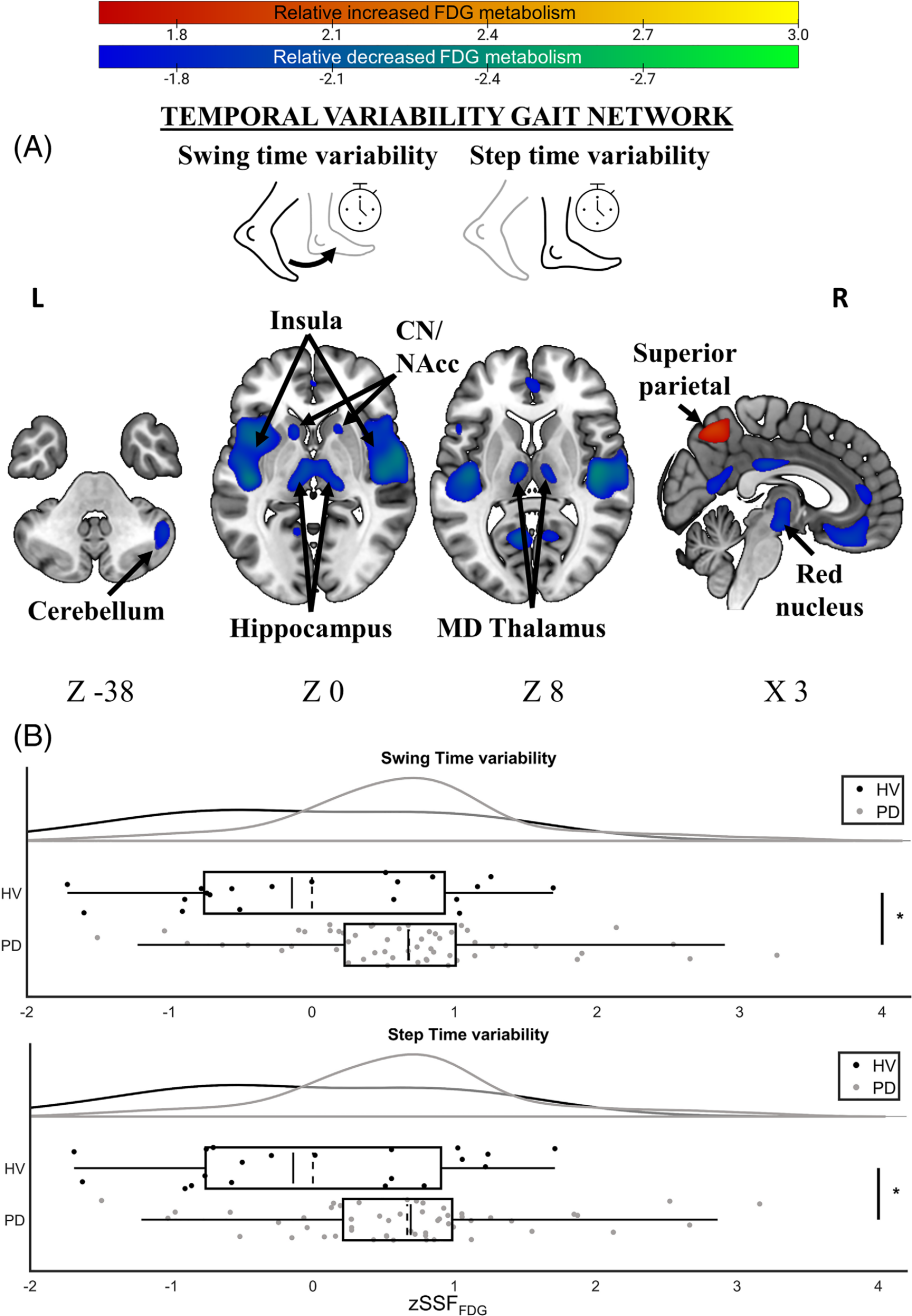
The temporal variability gait network. (**A**) The thresholded *z* voxel map is projected onto the ICBM152 template. Increased and decreased FDG metabolism is shown in hot and cold colors, respectively. (**B**) Distribution of *z* scored SSF_FDG_ for each group and discrete gait characteristics contained within this gait network. Greater zSSF_FDG_ (*x*‐axis) refers to greater network expression. *P < 0.05 corrected for multiple comparisons. Box and whiskers represent the interquartile range; solid line represents the median, while the broken line is the mean. CN, caudate nucleus; HV, healthy volunteers; MD, mediodorsal; NAcc, nucleus accumbens; PD, Parkinson's disease. zSSF_FDG_, standardized subject scaling factor. [Color figure can be viewed at wileyonlinelibrary.com]

*Swing time variability* (*R* ^*2*^ = 0.21, *P* = 0.003) and *step time variability* (*R* ^*2*^ = 0.12, *P* = 0.041) were predicted by a separate brain network that we refer to as a *temporal variability gait network* (Fig. 3A, linear combination of PCs 3 and 4). The topography of this covariance network was characterized by increased FDG metabolism in bilateral superior frontal and precentral gyri, right postcentral gyrus, mid‐cingulate, bilateral superior parietal lobules, precuneus, and superior occipital cortex with concurrently decreased metabolism in bilateral insula, hippocampus, calcarine gyrus, superior temporal gyri and mediodorsal thalamus, basal ganglia nuclei putamen and caudate, right red nucleus (RN), left nucleus accumbens (NAcc), and the right cerebellar Crus 1. Anatomical details of the *temporal variability gait network* are summarized in Supporting Information Table S2. Combined illustration of the two PDGP subnetworks is shown in Supporting Information Fig. S1.

Network expression was prospectively computed for HVs on a single‐case basis, to assess the ability of these two gait‐related metabolic networks to identify abnormal metabolic glucose in PD related to discrete gait impairments.^39^ Using multiple linear regression, results showed that network expression for both *pace gait network* (*mean step velocity*: B_GROUP_ = 0.67, *t*(69) = 2.29, *P* = 0.025, 95% confidence interval [CI] = [0.09, 1.25]; *mean step length*: B_GROUP_ = 0.63, *t*(69) = 2.15, *P* = 0.035, 95% CI = [0.05, 1.22]; Fig. 2B) and *temporal variability gait network* (*swing time variability*: B_GROUP_ = 0.54, *t*(69) = 2.32, *P* = 0.023, 95% CI = [0.07, 1.01]; *step time variability*: B_GROUP_ = 0.53, *t*(69) = 2.27, *P* = 0.027, 95% CI = [0.06, 1.00]; Fig. 3B) were significantly greater in participants with PD relative to HVs corrected for multiple comparisons. Regression diagnostics showed no evidence of collinearity among predictors (variance inflation factor ≅ 1), and no single case was overly influential on the models' ability to predict all cases (Cook's *d* < 1).

Investigating the relationship between the prospectively computed SSF_FDG_ for HVs and discrete gait characteristics showed nonsignificant associations for all (maximum |*r*| = 0.18, *P* = 0.44).

### 3.4 Relation Between PD Resting Gait Network Expression and Non–Gait‐Related Clinical Data

The relationship between SSF_FDG_ for discrete gait characteristics contained within PDGPs and other, non–gait‐related clinical data, including disease duration (time from diagnosis), LEDD, total MDS‐UPDRS III motor severity scores, and anticholinergic burden, in participants with PD indicated no statistically significant associations (maximum |*r*| = 0.20, *P* = 0.16; see Supporting Information Fig. S2).

## 4 Discussion

In this study, we used a comprehensive set of gait data and computed 12 discrete gait characteristics from a large group of participants with early‐stage PD and age‐matched HVs. This was combined with measures of metabolic glucose utilization acquired using resting FDG‐PET images that were interrogated with a multivariate spatial covariance approach. Our primary aim was to quantify novel discrete gait‐related metabolic covariance networks, characterize their topology, and explore if people with early‐stage PD express these networks differently than HVs.

Our results show that discrete gait characteristics, which are altered in PD, form two primary metabolic gait‐related brain networks (PDGPs) associated with numerous widespread brain regions, including those subserving motor and multisensory functions. Our findings additionally showed that expression of these PDGPs was abnormal in participants with PD, but that expression was not related to PD motor severity, LEDD, disease duration, or anticholinergic burden.

### 4.1 A Resting Pace Gait Network

Both mean gait velocity and mean step length formed a unique covariant metabolic network composed of regional covariance patterns from PCs 2 and 3. We termed this the *pace gait network* where a pattern of relatively increased and decreased FDG metabolism is associated with faster gait velocity and longer steps. The topography of this network was predominantly characterized by covariant increased metabolism in the DLPFC and SMA, both of which are part of the indirect locomotor pathway.^40^ Clusters including the SMA, which is consistently activated across studies of gait anatomy in PD,^41^ were also noted in the temporal variability network, indicating that the SMA may play a role in determining both gait velocity and variability in PD. To that end, increased metabolism in the SMA may lead to increased gait speed but at the cost of greater gait variability.

Relatively increased metabolism in the DLPFC and SMA covaried with the bilateral insula. The insula (which featured in both gait networks) is a highly versatile brain region, but its function in gait is unclear, and previous investigations on the role it plays in gait in PD are narrowly focused on patients with freezing of gait. Thus, we can only speculate on its precise role in our gait networks. The insula and the anterior subcentral sulcus in the right hemisphere are part of the vestibular system, which interprets information about spatial orientation and posture.^42^ Perturbations to this system may result in spatial disorientation and falls.^43^, ^44^ Furthermore, the insula may play a role in multimodal processing, such as mediating cognitive flexibility.^45^ Insula involvement may therefore suggest that increased gait velocity in PD is mediated by increased metabolism in both cognitive and motor regions. This notion is further strengthened by the increased FDG metabolism noted in a cluster encompassing the OFC and gyrus rectus, which are part of the frontoparietal network. Increased dopamine concentration in the OFC can both enhance and worsen different executive functions in PD,^46^ while reduced dopamine levels in the OFC increase cadence.^47^ One study showed that after withholding dopamine medication, participants with PD recruited nonmotor regions implicated in cognitive control during gait, whereas those *on* dopamine medication recruited regions implicated in motor control.^48^ In addition, the covariant negative‐weighted metabolism converged on temporal regions, including the fusiform gyrus. Its involvement in this gait network is corroborated by previous studies showing increased activity in this region in healthy adults during both real^49^ and imagined^50^ locomotion.

Subsequent analyses showed that people with PD express this network more strongly after controlling for confounding factors. Recent studies show that people with PD walk more slowly and with shorter steps than healthy older adults.^1^, ^2^ Inspection of Fig. 2B shows that some HVs express this particular PDGP more than patients with PD. This is in line with the work of others who suggest that a decline in these particular gait parameters is related to a combination of aging and PD‐related pathology.^3^ This may also explain why we did not observe a statistical relationship between expression of this network and disease duration in PD.

### 4.2 A Resting Temporal Variability Gait Network

Both swing time variability and step time variability formed another unique covariant metabolic network composed of regional covariance patterns from PCs 3 and 4. We termed this the *temporal variability gait network* where a pattern of relatively increased and decreased FDG metabolism is associated with greater (worse) gait variability. The topography of this network was characterized principally by reduced metabolism in the basal ganglia (right caudate and putamen and left NAcc) and temporal lobe, insula, and hippocampus in addition to the mediodorsal thalamus and cerebellum. The cerebellum, which was associated with both gait networks, has widespread connections with these cortical and basal ganglia nuclei forming the brain motor system.^51^ This relatively reduced FDG metabolism in the cerebellum and motor regions is consistent with findings in PD during free and repetitive motor execution,^52^ treadmill walking,^53^ and gait imagery.^54^ By contrast, cerebellar hyperactivity is observed during automatic motor control tasks in conjunction with hyperactive (pre)motor cortices, which is postulated to be a neural adaption in response to defective basal ganglia function.^55^, ^56^ Our findings are not inconsistent with this view.

We furthermore noted decreased metabolism in the hippocampus and the superior temporal gyri. The structural integrity of the hippocampus has been associated with altered gait velocity and step variability in healthy older adults, as well as in neurodegenerative disorders.^16^, ^17^ Also, reduced metabolism in the caudate, putamen, and NAcc as a function of increased gait variability resonates with recent findings.^48^ When participants with PD perform a virtual reality gait task and depress foot pedals in an alternating fashion, increased functional connectivity between both the caudate and the putamen with the NAcc correlated with increasing step time variability.^48^

We observed increased FDG metabolism in the ventrolateral thalamus in the pace gait network (Fig. 2) and decreased metabolism in the RN in the temporal variability gait network (Fig. 3). Both regions are crucial relay stations in the dentato‐rubro‐thalamic‐cortical pathway, which supplies afferent projections to the motor cortex.^57^ The ventrolateral thalamus is particularly active during locomotion,^58^ but the role of the RN in the neuropathology of PD is unclear. Evidence from PD animal models suggests that RN lesions result in rhythmic locomotor abnormalities, but gait velocity is unaffected.^59^ It must, however, be noted that these findings are interpreted with caution because of the low spatial specificity of PET images.

### 4.3 Comparison with Other Reported PD Profiles

We used the same SSM/PCA approach used to generate both PD motor (PDRP) and cognitive profiles (PDCP).^19^, ^21^ Comparison of the PDRP^19^ and PDCP^21^ with our gait profiles demonstrates overlap of regions demonstrating covarying glucose consumption, including the motor cortex, posterior parietal areas, cerebellum, prefrontal cortex, and putamen.

The PDRP is formed by the first PC,^19^ and this PC does not feature in our PDGPs. For completeness, we assessed the correlation between SSF_FDG_ expression scores for PC1 and PD clinical data. These analyses showed nonstatistically significant relationships for all comparisons (results not shown). By contrast, Huang and colleagues'^21^ network underpinning cognitive dysfunction in PD (PDCP) was PC2. Given that gait and cognition are intricately linked in both aging and neurodegeneration,^60^, ^61^ it does not come as a surprise that gait speed and step length forming the *pace gait network* were related to the combination of PCs 2 and 3. It is noteworthy that Huang and colleagues'^21^ PDCP was identified first in patients with PD with reference values for the expression of the PDCP computed prospectively in HVs; in this study, we have taken a similar approach. There are, however, important methodological differences between those studies and this study. Disease duration was substantially greater in the previous studies (this study: 6 months; others: 8–12 years). Also, in the other two studies, patients with PD were scanned OFF DART, whereas in this study, patients were scanned ~1 hour after DART.

Unlike the PDRP, which correlates with PD motor severity,^19^, ^62^, ^63^ our exploratory correlation analysis showed that motor severity, LEDD, disease duration, and anticholinergic burden were not related to the expression of the PDGPs. This conforms with recent findings^3^, ^5^ and suggests that pathology‐related impairment in gait and postural control may be caused by nondopaminergic brain biochemistry.^40^ Indeed, our findings lend some credence to the notion that selective gait variability characteristics are susceptible to altered cholinergic function,^5^, ^9^ because the *temporal variability gait network* was associated with FDG hypermetabolism and hypometabolism in sensorimotor regions, caudate, RN (containing choline acetyltransferase), and hippocampus. These regions receive dense cholinergic innervation from the nucleus basalis of Meynert and brainstem pedunculopontine nucleus.^64^, ^65^ Alterations in these regions are likely contributors to functional decline in cognition and increased falls risk.^61^ In support of this, a recent structural imaging study from our group showed that atrophy of the nucleus basalis of Meynert in PD is a strong predictor of a progressive decline in gait variability.^9^

Expression of the PDRP increases in the advancing stages of the disease but can be readily reversed by both levodopa infusion and subthalamic nucleus deep brain stimulation.^66^, ^67^ Novel noninvasive electrical stimulation of the cholinergic vagus nerve is being assessed to mitigate gait impairments in PD,^68^, ^69^, ^70^ showing promising success in improving step time variability.^69^ To that end, we speculate whether our PDGPs may be considered as a conceptual framework for future studies targeting dopaminergic treatment–resistant gait characteristics. Future studies could assess, similar to Huang and colleagues,^21^ whether the expression of PDGPs can be readily reversed by interventions and whether reduced expression of these networks coincides with improvement in gait characteristics contained within them.

### 4.4 Study Strengths and Limitations

The main strength of our study is the exploration of a comprehensive set of gait characteristics interrogating a relatively large group of both participants with PD and HVs to quantify discrete neural networks of gait mapping to distinct outcomes. This dataset provided a unique opportunity to correlate resting glucose metabolic changes in brain networks with discrete gait impairments because of pathology allowing specific differences to be explored. Understanding the pathophysiological signatures of gait problems in PD is of the highest priority because DART to mitigate these are limited. The main limitations of this study are that FDG‐PET scans were acquired at rest, and this might not give the most accurate representation of brain metabolism during locomotion. Imaging the patient cohort with FDG‐PET after taking their regular PD medication is in contrast with current practice^71^ and a possible limitation of our methodology. This approach will have suppressed the PDRP, although it did not prevent extraction of PDGPs for the PD cases. These PDGPs will have reflected a contribution of nondopaminergic rather than dopaminergic components, although this requires further validation in a larger cohort of treated and nontreated patients. The inclusion of patients on anticholinergic medication may seem counterproductive to our speculation that parts of the PDGPs may be cholinergically mediated. However, we note that all patients were on low doses and their individual network expression scores do not indicate that they were influential. A nonstatistical relationship between ADS and SSF_FDG_ is evidence for this assertion. Finally, the absence of anatomical images precludes controlling for interindividual variability in gray matter atrophy.

## 5 Conclusions and Future Work

We have characterized altered networks of FDG metabolism across voxels in higher‐order cognitive, frontoparietal network, multisensory, motor control, and cerebellar brain regions, which are related to discrete gait difficulties in early‐stage PD forming distinct PDGPs. Our results suggest that unique pathophysiological signatures of gait impairments and postural instability exist in PD. These gait networks may potentially function as compensatory mechanisms to improve gait control to counteract striatal dopamine denervation and aberrant cholinergic function in PD. These networks may hold potential as neuroimaging biomarkers for early disease identification, facilitating effective interventions to mitigate mobility decline and risk of falls. Future studies should assess the stability and reliability of these gait networks with a longitudinal design and multimodal imaging battery. In addition, future studies with a larger sample size could consider a comparison between PD motor phenotypes to assess whether these types of network are specific to clinically defined gait‐related motor impairment while keeping in mind that phenotype status is variable in the early stages of the disease.^72^ This may lead to further understanding of the mechanisms underpinning PD gait‐related changes.

## Author Roles

1. Research Project: A. Conception, B. Organization, C. Execution;

2. Statistical Analysis: A. Design, B. Execution, C. Review and Critique;

3. Manuscript Preparation: A. Writing the First Draft, B. Review and Critique.

H.P.S.: 2A, 2B, 3A, 3B

A.J.Y.: 1B, 1C, 3B

B.G.: 1C, 2C, 3B

S.L.: 1B, 1C, 3B

L.A.: 1C, 3B

R.A.L.: 1C, 2C, 3B

S.J.C.: 2A, 2B, 2C, 3B

M.J.F.: 1B, 1C, 3B

J.‐P.T.: 2A, 2C, 3B

N.P.: 2C, 3B

D.J. Brooks: 2C, 3B

J.T.O.: 1A, 3B

D.J. Burn: 1A, 3B

L.R.: 1A, 1B, 1C, 2C, 3B

## Financial Disclosures

This work was supported by grants from Parkinson's UK (J‐0802, G‐1301) and Lockhart Parkinson's Disease. The work has been supported by the National Institute for Health Research (NIHR) Biomedical Research Unit based at Newcastle upon Tyne Hospitals National Health Service Foundation Trust and Newcastle University; the NIHR Newcastle BRC; and Newcastle CRF Infrastructure funding.

H.P.S.'s salary is paid by grants from Parkinson's UK and Dunhill Medical Trust awarded to A.J.Y., who is additionally supported by the Newcastle NIHR BRC plus grants from The Michael J. Fox Foundation, EU IMI, NIHR, Cure Parkinson's Trust, Lewy Body Society, Intercept Pharmaceuticals, and the Weston Brain Institute. She has received honoraria from Teva‐Lundbeck, GE Healthcare, Bial and Parkinson's Academy and sponsorship from Teva‐Lundbeck, UCB, GlaxoSmithKline, Genus, Britannia, and AbbVie for attending conferences. B.G., S.L., and D.J. Burn: none.

L.A.'s salary is supported by the NIHR Newcastle Clinical Research Facility (CRF) infrastructure, and L.A. has received grants from the Economic and Social Research Council (ESRC), Medical Research Council (MRC), Parkinson's UK, and the Royal College of Radiologists.

R.A.L. is supported by a Janet Owens Parkinson's UK Senior Research Fellowship (F‐1801) and has received funding grants from the Lewy Body Society, MRC Discovery Medicine North (DiMeN) Doctoral Training Partnership, and NIHR Newcastle Biomedical Research Centre.

S.J.C. and M.J.F. are supported by Newcastle NIHR BRC.

J.‐P.T. is supported by Newcastle NIHR BRC. He has received speaker fees from GE Healthcare, has received funding from Sosei‐Heptares, and has consulted for Kyowa‐Kirin.

N.P. has received grants from the Independent Research fund in Denmark, Danish Parkinson's disease Association, Parkinson's UK, Center of Excellence in Neurodegeneration (CoEN) network award, GE Healthcare, Multiple System Atrophy Trust, Weston Brain Institute, EU Joint Program Neurodegenerative Disease Research (JPND), EU Horizon 2020 research and innovation programme, and the Italian Ministry of Health.

D.J. Brooks has received grants from the Danish Council for Independent Research, Lundbeck Foundation, Alzheimer Research Trust, Horizon 2020 AD Detect and Prevent with Oxford and Nottingham Universities and Brain+, Eurostars/Danish Innovation Fund Alzheimer Shield with Karolinska Institute and Brain+, and GE Educational grant. He has also received consultancy fees from Biogen.

J.T.O. has research programs supported by the Alzheimer's Society, the Alzheimer's Association, the MRC, the NIHR Cambridge Biomedical Research Centre, Merck, and Alliance Medical. He has acted as a consultant for Biogen, TauRx, Eisai, Nova Nordisk, and GE Healthcare.

L.R.'s research program is supported in part by grants from the MRC, European Union, Parkinson's UK, NIHR BRC, Cure Parkinson's Trust, Health Research Council New Zealand, Dunhill Medical, and Stroke Association.

## Supporting information


**Table S1. Pace gait network.** Clusters anatomical regions, associated Z‐statistic and MNI coordinates showing relative increased and decreased FDG metabolism. Clusters are labelled according to the AAL atlas.
**Table S2. Temporal variability gait network.** Clusters anatomical regions, labels, associated Z‐statistic and MNI coordinates showing relative increased and decreased FDG metabolism. Clusters are labelled according to the AAL atlas.
**Figure S1. Combined image of the Parkinson's Disease Gait‐related Patterns (PDGP's).** Subnetworks of the PDGP's are projected onto both surface and volume templates.
**Figure S2. Clinical correlations.** Partial correlations between residualised network expression and clinical data in Parkinson's. Correlation of clinical data with step velocity and step length (pace gait network) are shown in columns 1 and 2, respectively. Correlation of clinical data with swing time variability (SD) and step time variability (SD) (temporal variability gait network) are shown in columns 3 and 4, respectively. Note, x‐axis label is consistent across all subplots.

## Data Availability

Data will be made available to bona fide academic researchers on reasonable request.
