# Supplementary material for: Gait‐Related Metabolic Covariance Networks at Rest in Parkinson's Disease
Source: Mov Disord. 2022 Mar 14;37(6):1222–34. doi: 10.1002/mds.28977 (PMC9314598; doi:10.1002/mds.28977)
Supplement: Supplementary file 1 — Table S1. Pace gait network. Clusters anatomical regions, associated Z‐statistic and MNI coordinates showing relative increased and decreased FDG metabolism. Clusters are labelled according to the AAL atlas. Table S2. Temporal variability gait network. Clusters anatomical regions, labels, associated Z‐statistic and MNI coordinates showing relative increased and decreased FDG metabolism. Clusters are labelled according to the AAL atlas. Figure S1. Combined image of the Parkinson's Disease Gait‐related Patterns (PDGP's). Subnetworks of the PDGP's are projected onto both surface and volume templates. Figure S2. Clinical correlations. Partial correlations between residualised network expression and clinical data in Parkinson's. Correlation of clinical data with step velocity and step length (pace gait network) are shown in columns 1 and 2, respectively. Correlation of clinical data with swing time variability (SD) and step time variability (SD) (temporal variability gait network) are shown in columns 3 and 4, respectively. Note, x‐axis label is consistent across all subplots. [file MDS-37-1222-s001.docx]

Supplementary material to:

**Gait-related metabolic covariance networks at rest in Parkinson’s disease**

Hilmar P. Sigurdsson, Alison J. Yarnall, Brook Galna, Sue Lord, Lisa Alcock, Rachael A Lawson, Sean J. Colloby, Michael J. Firbank, John-Paul Taylor, Nicola Pavese, David J. Brooks, John T. O'Brien, David J. Burn, Lynn Rochester

Correspondence: Hilmar P. Sigurdsson (hilmar.sigurdsson@newcastle.ac.uk)

**Table S1. Pace gait network.** Clusters anatomical regions, associated Z-statistic and MNI coordinates showing relative increased and decreased FDG metabolism. Clusters are labelled according to the AAL atlas.

| AAL label | Side | Z-statistic |  | MNI |  |
| --- | --- | --- | --- | --- | --- |
|  |  |  | x | y | z |
| **Increased FDG** |  |  |  |  |  |
| Anterior cingulate cortex, superior | L | 1.92 | -6 | 34 | 23 |
| Mid cingulate cortex | L | 2.03 | -6 | -27 | 34 |
| Posterior cingulate cortex | R | 1.85 | 0 | -51 | 31 |
| Inferior frontal gyrus, opercular part | R | 2.32 | 48 | 16 | 26 |
| Inferior frontal gyrus, orbital part-2 | L | 2.59 | -36 | 24 | -5 |
| Inferior frontal gyrus, triangular part | L | 2.04 | -46 | 29 | 23 |
| Middle frontal gyrus-2 | L | 2.52 | -30 | 39 | 31 |
| Middle frontal gyrus-2 | R | 2.41 | 48 | 42 | 15 |
| Superior frontal gyrus-2 | L | 2.42 | -26 | 58 | 11 |
| Superior frontal gyrus-2 | R | 2.60 | 26 | 62 | 5 |
| Superior frontal gyrus, medial | R | 2.47 | 6 | 48 | 5 |
| Heschl gyrus | L | 2.24 | -42 | -20 | 10 |
| Insular cortex | L | 2.36 | -38 | -14 | -2 |
| Insular cortex | R | 2.84 | 38 | 20 | -4 |
| Orbital frontal cortex-posterior | L | 2.61 | -34 | 22 | -15 |
| Postcentral gyrus | R | 1.88 | 62 | -16 | 15 |
| Precentral gyrus | L | 1.77 | -40 | 5 | 40 |
| Rectus gyrus | R | 2.48 | 2 | 46 | -19 |
| Supplementary motor area | L | 1.85 | -14 | 17 | 64 |
| Middle temporal gyrus | L | 1.84 | -50 | 4 | -20 |
| Superior temporal pole | R | 1.75 | 38 | 23 | -32 |
| Superior temporal gyrus | R | 2.07 | 50 | -28 | 9 |
| Thalamus, ventrolateral | R | 1.90 | 8 | -8 | 6 |
|  |  |  |  |  |  |
| **Decreased FDG** |  |  |  |  |  |
| Calcarine gyrus | R | -2.37 | 22 | -98 | -6 |
| Cerebellum lob. 4,5 | R | -1.71 | 36 | -29 | -33 |
| Cerebellum lob. 6 | L | -1.78 | -32 | -34 | -34 |
| Cerebellum lob. 6 | R | -2.04 | 12 | -80 | -17 |
| Cerebellum lob. 8 | L | -1.89 | -22 | -55 | -47 |
| Cerebellum lob. 9 | L | -1.90 | -10 | -45 | -47 |
| Cerebellum Crus 1 | L | -2.18 | -18 | -90 | -24 |
| Cuneus | L | -1.97 | -13 | -85 | 35 |
| Cuneus | R | -2.14 | 17 | -83 | 34 |
| Fusiform gyrus | L | -2.03 | -22 | 3 | -40 |
| Fusiform gyrus | R | -2.21 | 36 | -41 | -23 |
| *Continue on next page* |  |  |  |  |  |
| Lingual gyrus | R | -2.40 | 20 | -88 | -8 |
| Middle occipital cortex | L | -2.30 | -22 | -92 | 2 |
| Paracentral lobule | L | -1.82 | -12 | -31 | 56 |
| Paracentral lobule | R | -1.83 | 16 | -33 | 51 |
| Middle temporal gyrus | L | -1.79 | -50 | -26 | -12 |
| Middle temporal pole | R | -1.79 | 30 | 5 | -36 |
| Abbr. lob – lobule, R – right, L – left. | | | | | |

**Table S2. Temporal variability gait network.** Clusters anatomical regions, labels, associated Z-statistic and MNI coordinates showing relative increased and decreased FDG metabolism. Clusters are labelled according to the AAL atlas.

| AAL label | Side | Z-statistic |  | MNI |  |
| --- | --- | --- | --- | --- | --- |
|  |  |  | x | y | z |
| **Increased FDG** |  |  |  |  |  |
| Precentral gyrus | L | 2.15 | -16 | -15 | 60 |
| Precentral gyrus | R | 1.96 | 20 | -21 | 62 |
| Superior frontal gyrus-2 | L | 1.71 | -16 | 11 | 50 |
| Superior frontal gyrus-2 | R | 1.76 | 16 | 33 | 41 |
| Middle frontal gyrus-2 | R | 1.70 | 36 | 9 | 40 |
| Superior occipital cortex | L | 1.85 | -11 | -79 | 41 |
| Superior occipital cortex | R | 1.87 | 25 | -77 | 43 |
| Postcentral gyrus | R | 1.94 | 34 | -39 | 65 |
| Superior parietal lobe | L | 1.87 | -24 | -57 | 55 |
| Superior parietal lobe | R | 2.32 | 16 | -51 | 53 |
| Inferior parietal lobe | R | 2.26 | 26 | -55 | 53 |
| Precuneus | L | 1.88 | -10 | -55 | 63 |
| Precuneus | R | 1.95 | 20 | -67 | 41 |
| Paracentral lobule | L | 1.72 | -16 | -27 | 74 |
|  |  |  |  |  |  |
| **Decreased FDG** |  |  |  |  |  |
| Anterior cingulate cortex, pre | R | -1.81 | 2 | 44 | 9 |
| Calcarine gyrus | L | -2.21 | -8 | -62 | 11 |
| Calcarine gyurs | R | -1.88 | 12 | -60 | 11 |
| Caudate nucleus | R | -1.87 | 16 | 16 | -2 |
| Cerebellum Crus 1 | R | -1.94 | 48 | -57 | -37 |
| Mid cingulate cortex | R | -2.02 | 4 | -23 | 30 |
| Middle frontal gyrus-2 | L | -1.84 | -36 | 33 | 27 |
| Heschl gyrus | R | -2.35 | 46 | -14 | 8 |
| Hippocampus | L | -2.20 | -18 | -26 | -10 |
| Hippocampus | R | -2.09 | 18 | -24 | -6 |
| Insular cortex | L | -2.31 | -34 | 20 | -6 |
| *Continue on next page* |  |  |  |  |  |
| Insular cortex | R | -2.19 | 38 | 20 | -8 |
| Nucleus accumbens | L | -1.98 | -10 | 16 | -6 |
| Putamen | R | -1.75 | 26 | 8 | -6 |
| Rectus gyrus | R | -2.06 | 0 | 30 | -21 |
| Red nucleus | R | -1.99 | 6 | -14 | -12 |
| Superior temporal gyrus | L | -2.30 | -44 | -20 | 0 |
| Superior temporal gyrus | R | -2.30 | 60 | -4 | 2 |
| Thalamus, medio-dorsal | L | -2.14 | -8 | -14 | 6 |
| Thalamus, medio-dorsal | R | -2.03 | 8 | -12 | 4 |
| Abbr. R – right, L - left | | | | | |


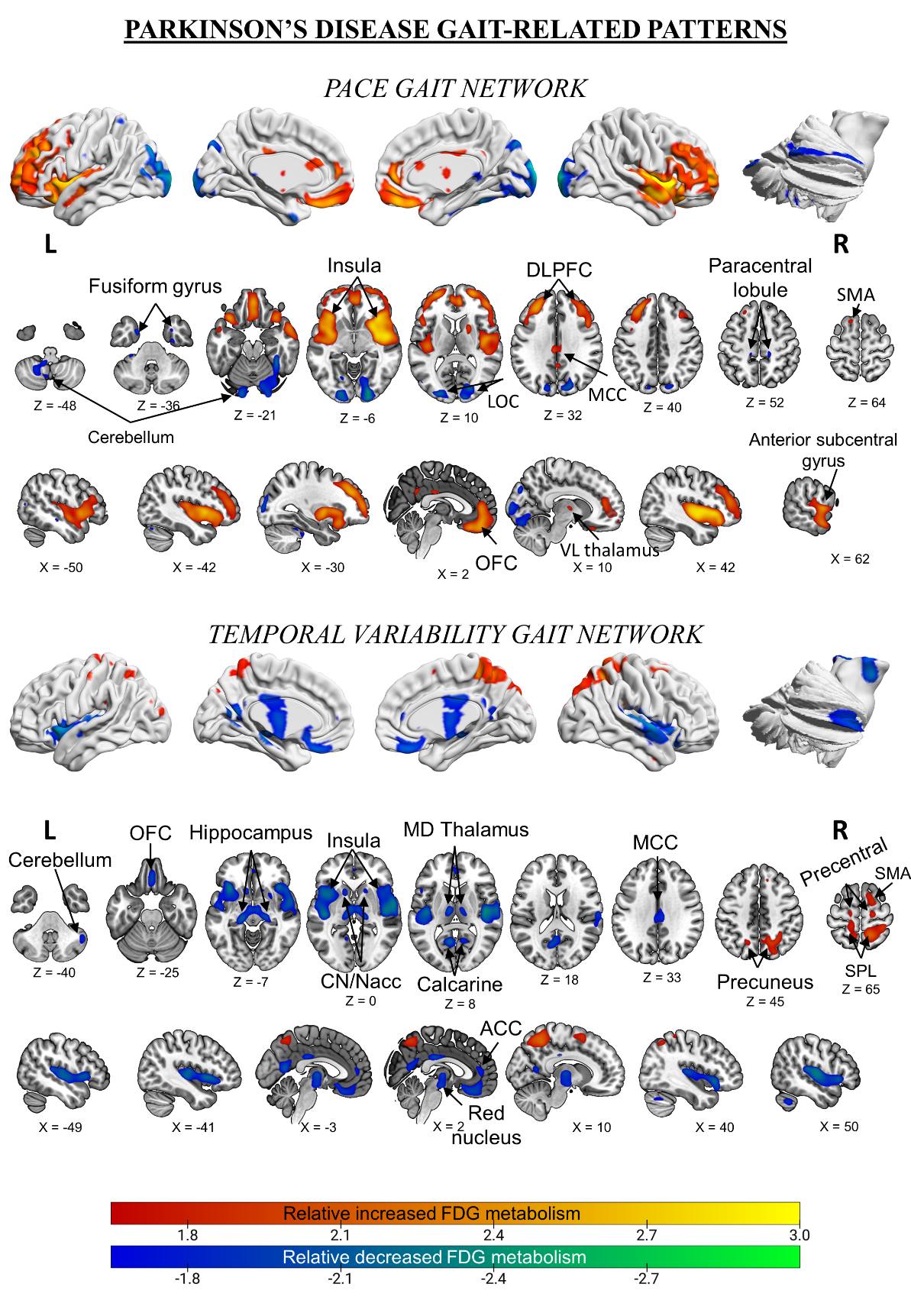


**Figure S1. Combined image of the Parkinson’s Disease Gait-related Patterns (PDGP’s).** Subnetworks of the PDGP’s are projected onto both surface and volume templates.


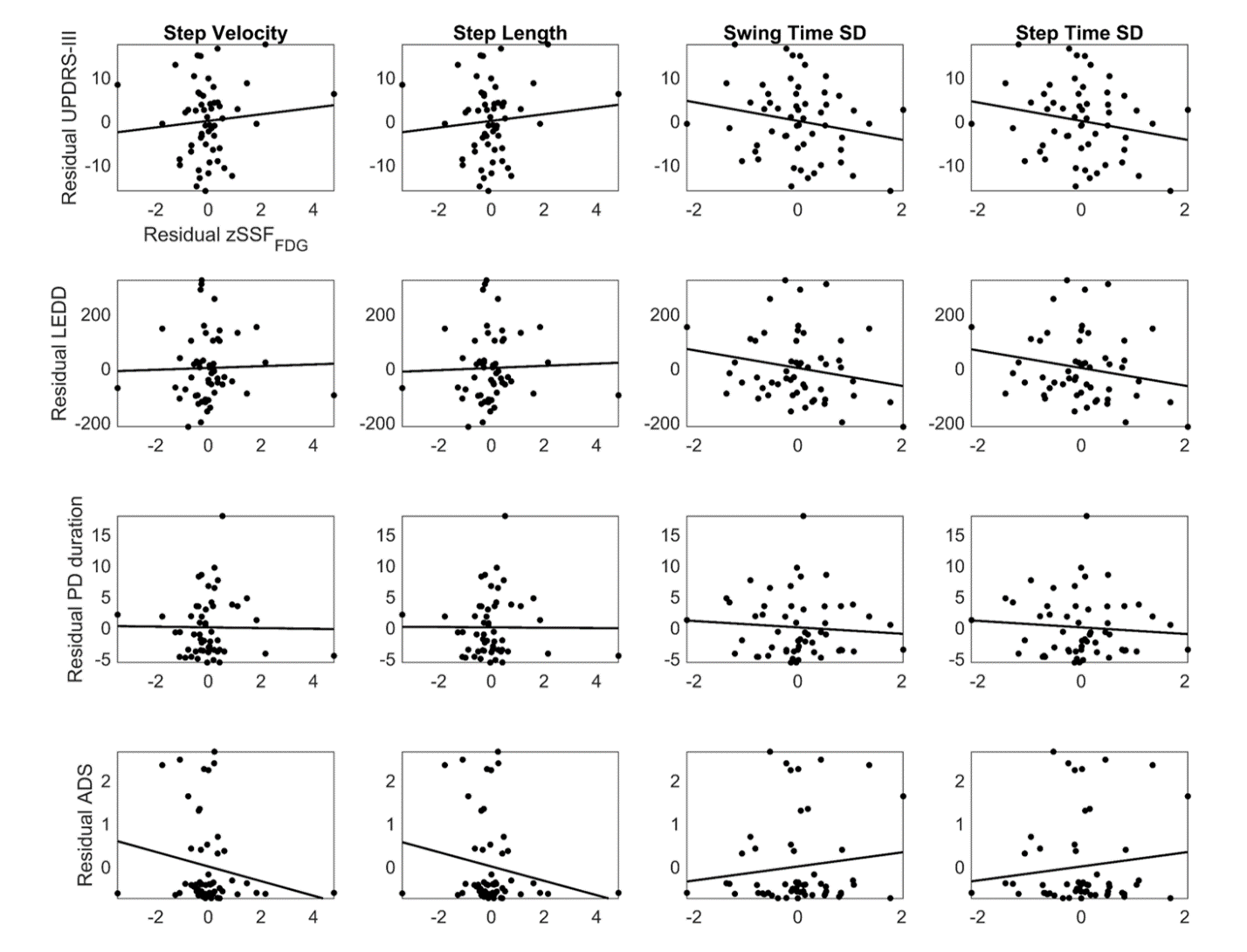


**Figure S2. Clinical correlations.** Partial correlations between residualised network expression and clinical data in Parkinson’s. Correlation of clinical data with step velocity and step length (pace gait network) are shown in columns 1 and 2, respectively. Correlation of clinical data with swing time variability (SD) and step time variability (SD) (temporal variability gait network) are shown in columns 3 and 4, respectively. Note, x-axis label is consistent across all subplots.
